# Planned private homebirth in Victoria 2000–2015: a retrospective cohort study of Victorian perinatal data

**DOI:** 10.1186/s12884-018-1996-6

**Published:** 2018-09-04

**Authors:** Miranda L. Davies-Tuck, Euan M. Wallace, Mary-Ann Davey, Vickie Veitch, Jeremy Oats

**Affiliations:** 1 The Ritchie Centre, Hudson Institute of Medical Research, 27-31 Wright Street, Clayton, Vic, 3168 Australia; 2 Safer Care Victoria, 50 Lonsdale Street, Melbourne, 3000 Australia; 3 Department of Obstetrics and Gynaecology, School of Clinical Sciences, Monash University, 246 Clayton Rd, Clayton, Vic, 3168 Australia; 4 Consultative Council on Obstetric and Paediatric Mortality and Morbidity (CCOPMM) Department of Health and Human Services, 50 Lonsdale Street, Melbourne, 3000 Australia

**Keywords:** Home birth, High risk, Perinatal outcomes

## Abstract

**Background:**

The outcomes for planned homebirth in Victoria are unknown. We aimed to compare the rates of outcomes for high risk and low risk women who planned to birth at home compared to those who planned to birth in hospital.

**Methods:**

We undertook a population based cohort study of all births in Victoria, Australia 2000–2015. Women were defined as being of low or high risk of adverse pregnancy outcomes according to the eligibility criteria for homebirth and either planning to birth at home or in a hospital setting at the at the onset of labour. Rates of perinatal and maternal mortality and morbidity as well as obstetric interventions were compared.

**Results:**

Three thousand nine hundred forty-five women planned to give birth at home with a privately practising midwife and 829,286 women planned to give birth in a hospital setting. Regardless of risk status, planned homebirth was associated with significantly lower rates of all obstetric interventions and higher rates of spontaneous vaginal birth (*p* ≤ 0.0001 for all). For low risk women the rates of perinatal mortality were similar (1.6 per 1000 v’s 1.7 per 1000; *p* = 0.90) and overall composite perinatal (3.6% v’s 13.4%; *p* ≤ 0.001) and maternal morbidities (10.7% v’s 17.3%; *p* ≤ 0.001) were significantly lower for those planning a homebirth. Planned homebirth among high risk women was associated with significantly higher rates of perinatal mortality (9.3 per 1000 v’s 3.5 per 1000; *p* = 0.009) but an overall significant decrease in composite perinatal (7.8% v’s 16.9%; *p* ≤ 0.001) and maternal morbidities (16.7% v’s 24.6%; *p* ≤ 0.001).

**Conclusion:**

Regardless of risk status, planned homebirth was associated with significantly lower rates of obstetric interventions and combined overall maternal and perinatal morbidities. For low risk women, planned homebirth was also associated with similar risks of perinatal mortality, however for women with recognized risk factors, planned homebirth was associated with significantly higher rates of perinatal mortality.

## Introduction

Only 1 in 200 women have a homebirth in Victoria, Australia. This is despite international evidence that for healthy normal pregnant women, home birth is not associated with an increased rate of adverse perinatal outcomes [1–7], or maternal morbidity [1, 3–8] compared to similar women having a planned hospital birth, particularly if they are multiparous [9]. Whether homebirth is safe for women at high risk of adverse pregnancy outcomes is less clear. Findings from studies in populations which were mixed with regards to risk status report higher rates of adverse perinatal outcomes compared to similar women in hospital [10, 11]. In contrast those that specifically looked at high risk women, identified a reduction in adverse outcomes and obstetric interventions [12] or no difference (apart from a higher vaginal birth rate) in outcomes [13]. Homebirth in England however is more common and integrated into the health system. Whether these findings from England apply to Australia is not clear.

In 2013 a perinatal death occurred in Victoria during a planned homebirth in a woman who was identified as being at high risk of adverse pregnancy outcomes. Following the investigation of this case a coronial recommendation (Coroner Parkinson 2013) was made for the Health Minister to investigate and provide information about the rates of outcomes for planned homebirth in Victoria. This research directly addresses the coroner’s recommendation.

We aimed to quantify the number of women who planned to birth at home in Victoria between 2000 and 2015, to quantify transfer rates to hospital, and to compare the rates of perinatal and maternal outcomes and obstetric interventions for women who planned to birth at home compared to those who planned to birth within a hospital setting by maternal ‘risk’ status.

## Methods

We undertook a study using data that are legislatively routinely reported on all births in Victoria, Australia to the Victorian Perinatal Data Collection (VPDC). For every birth ≥20 weeks gestation(or ≥ 400 g birth weight if gestation not known), regardless of place of birth the VPDC receives a standardized report detailing over 100 items regarding maternal characteristics, obstetric conditions, procedures and outcomes, perinatal mortality and morbidity and birth defects.

De-identified data for all births from ≥37 weeks gestational age from 2000 to 2015 were extracted. Babies with congenital anomalies or where a caesarean birth was planned were excluded. The majority of planned home births in Victoria occur under the care of a privately practising midwife. Two small public homebirth programs, caring for about 90 women a year in total, were established in 2014. Due to the small numbers and because they have only been operating for two of the 16 years studied, we excluded them from analyses. Women were identified as being of low or high risk informed by the Australian College of Midwives(ACM) guidelines for consultation and referral [14]. These are the criteria referenced in public funded homebirth programs to inform eligibility for planned homebirth.

Specifically, a high risk pregnancy was defined as: a multiple pregnancy, a post-term (> 41 + 6 weeks of gestation) pregnancy, a non-cephalic presentation in labour, obesity(BMI Class 2 or greater: data only available from 2009 onwards), a prior caesarean, previous uterine surgery, grand multiparity(≥5 previous births), any significant maternal medical condition such as pre-existing diabetes, hypertension, renal, cardiac, liver, respiratory, endocrine, immunological, renal, or gastrointestinal disease as determined by individual ICD-10 codes. All other women were classified as having a low risk pregnancy.

Women were grouped by their planned place of birth at the beginning of labour. This was done by utilising the VPDC fields planned place of birth, changed intent of planned place of birth, and timing (antepartum/intrapartum) of changed intent. Additional checks were done to determine who reported the birth also, specifically looking for the codes that identified privately practising midwives*.* The planned hospital group also includes birth centre births. For the period of this study however all birth centre births were integrated within a larger hospital health service.

Data were largely complete. Where data were missing a ‘not reported’ variable was used. BMI data were only available from 2009 onwards. Validation of the accuracy of the dataset has been reported [15]. For the variables used in this study that have been validated (demographics, mortality outcomes and obstetric interventions), all had accuracy above 90% except for BMI.

### Statistical analyses

The number of women who, at the onset of labour planned to give birth at home were tabulated and graphed by risk status and overall by year. The characteristics of the women and the frequency of risk factors in the high risk pregnancy group were tabulated. Actual place of birth was used to determine transfer rates. The rates of perinatal and maternal outcomes and obstetric interventions by planned place of birth and risk status were determined using the Chi^2^ test. The denominator for perinatal outcomes was the number of babies born, for maternal outcomes and obstetric outcomes it was the number of women who gave birth. A small number, 0.4%, of planned hospital births are born before arrival but were not excluded from the dataset. All statistical analyses were undertaken using Stata/IC 12.1 for Mac (College Station, TX USA). A *p* value < 0.05(2-tailed) was regarded as statistically significant.

## Results

**Fig. 1:**
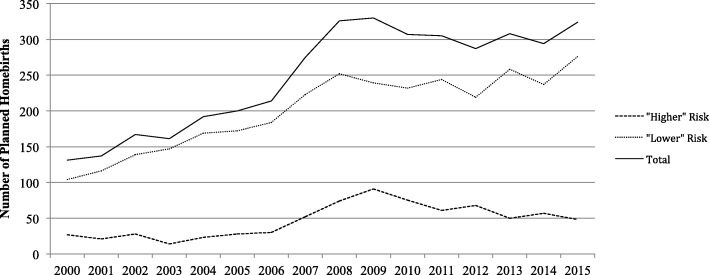
Number of women per annum who, at the beginning of labour, planned to birth at home with a privately practising midwife

From 2000 to 2015, 3945 women planned to give birth at home with a privately practising midwife and 829,286 women planned to give birth in hospital. Of those who planned to give birth at home 3202(81%) were identified as having a low risk pregnancy and 743(19%) were identified as having a high risk pregnancy. Of those planning a hospital birth 701,058(85%) were identified as low risk and 128,228(15%) were identified as high risk. The number of women planning a homebirth, regardless of risk status, increased from 131 per annum in 2000 to 315 per annum in 2015. This reflected a 2.6 fold and 1.8 fold increase for low risk and high risk women, respectively (Fig. 1). The characteristics of the women are presented in Table 1. Regardless of risk status, women who planned a homebirth were older, had a normal BMI, were Australian-born, were parous and were of higher socioeconomic status compared to their equivalent group who planned to give birth in hospital. Of the 3202 low risk and 743 high risk mother-baby pairs who planned a home birth, 2888(90%) and 628(83%) gave birth at home.

**Table 1:** Characteristics of women planning give birth at home in Victoria (2000–2015)

	Low risk		High risk	
	Planned home birth *n* = 3202	Planned hospital birth *n* = 701,058	Planned home birth *n* = 743	Planned hospital birth *n* = 128,228
Maternal Age				
< 20 yrs	19 (0.6%)	21,845 (3.1%)	2 (0.3%)	2051 (1.6%)
20-30 yrs	870 (27.2%)	292,891 (41.8%)	162 (21.8%)	44,543 (34.7%)
30 yrs plus	2269 (70.9%)	385,833 (55%)	573 (77.1%)	81,605 (63.6%)
Missing	44 (1.4%)	489 (0.1%)	6 (0.8%)	29 (0.02%)
Body Mass Index^a^				
< 18.5	74 (2.3%)	9642 (1.4%)	15 (2.0%)	1310 (1.0%)
18.5 to 24.9	902 (28.2%)	155,936 (22.2%)	196 (26.4%)	25,437 (19.8%)
25–29.9	284 (8.9%)	74,788 (10.7%)	85 (11.4%)	16,288 (12.7%)
30plus	98 (3.1%)	30,515 (4.4%)	73 (9.8%)	32,406 (25.3%)
missing	1844 (57.6%)	430,177 (61.4%)	374 (50.4%)	52,788 (41.2%)
Country of Birth				
Australian/NZ	2515 (78.5%)	500,945 (71.5%)	606 (81.6%)	87,369 (68.1%)
Non-Australian	481 (15.0%)	175,420 (25.0%)	91 (12.3%)	35,301 (27.5%)
Missing	206 (6.4%)	24,693 (3.5%)	46 (6.2%)	5558 (4.3%)
Parity				
Nulliparous	1103 (34.5%)	347,079 (49.5%)	142 (19.1%)	41,499 (32.4%)
Second birth	1187 (37.1%)	218,790 (31.2%)	294 (39.6%)	48,341 (37.7%)
3rd or subsequent birth	912 (82.5%)	135,181(19.3%)	306 (41.2%)	36,829 (28.7%)
Missing	0 (0%)	8 (0.001%)	1 (0.1%)	1559 (1.2%)
IRSD^b^				
1 Most Disadvantaged	365 (11.4%)	130,638 (18.6%)	84 (11.3%)	29,013 (22.6%)
2	544 (17.0%)	130,782 (18.7%)	110 (14.8%)	26,024 (10.3%)
3	567 (17.7%)	135,146 (19.3%)	134 (18.0%)	23,966 (18.7%)
4	740 (21.1%)	132,770 (18.9%)	177 (23.8%)	21,320 (16.6%)
5-Least Disadvantaged	846 (26.4%)	132,991(19.0%)	176 (23.7%)	19,244 (15.0%)
Missing	140 (4.4%)	38,731 (5.5%)	62 (8.3%)	8661 (6.8%)

Data presented as N (%)

^a^not collected until 2009

^b^Index of Relative Social Disadvantage (IRSD) quintile

Table 2 summarises the frequencies of risk factors for women with high risk pregnancies. Among women who planned to birth at home, 75(10%) had more than one risk factor and among the planned hospital group 16,438(12.5%) did. The most common risk factors for those who planned to birth at home were having had a previous caesarean birth (41.9%) and being post term (32.5%). For high risk women who planned to give birth in hospital having a pre-existing medical condition was the most common risk factor (31%) followed by having had a previous caesarean delivery (28.8%).

**Table 2:** Risk Factors among high risk mother-baby pairs

	Planned home birth *n* = 755	Planned hospital birth *n* = 131,727
Multiple Pregnancy	24 (3.2%)	6940 (5.3%)
Post-term Gestation	245 (32.5%)	11,438 (8.7%)
Non-vertex presentation	81 (10.7%)	18,129 (14.0%)
Body Mass Index > = 35	47 (6.2%)	22,347 (17.0%)
Previous Caesarean	316 (41.9%)	37,963 (28.8%)
Grand-multiparous	74 (9.8%)	10,562 (8%)
Maternal Medical Condition	42 (5.6%)	40,786 (31.0%)

Data presented as Number and %

Some women had more than one risk factor present

### Perinatal outcomes (Table 3)

There were no statistically significant differences in the stillbirth and neonatal mortality rates, low 5 min Apgar score, HIE or other perinatal morbidities between low risk women who planned to give birth at home compared to those who planned to give birth in hospital. The rate of admission to NICU was significantly higher in the planned homebirth group (0.44% v’s 0.20%, *p* = 0.03), while rates of admission to SCN(1.8% v’s to 8.3%, *p* < 0.001), birth trauma(1.4% v’s 6.6%, *p* < 0.001), intrauterine hypoxia(1.5% v’s 5.8%, *p* < 0.001) and the composite perinatal morbidity(3.6% v’s 13.4%, *p* < 0.001) were significantly lower.

**Table 3:** Rates of adverse perinatal outcomes among planned home and hospital births

	Planned low risk at home	Planned low risk in hospital	*p* Value
	*n* = 3202	*n* = 701,058	
Stillbirth	2 (0.62 per 1000)	906 (1.29 per 1000)	0.29
Neonatal death	3 (0.94 per 1000)	262 (0.37 per 1000)	0.10
Perinatal Mortality	5 (1.6 per 1000)	1168 (1.7 per 1000)	0.90
Admission to SCN	58 (1.8%)	58,303 (8.3%)	< 0.001
Admission to NICU	14 (0.4%)	1721 (0.2%)	0.03
Apgar< 7 @ 5 min	29 (0.9%)	8739 (1.2%)	0.08
HIE	0 (0%)	133 (0.2%)	0.44
Birth Trauma^a^	46 (1.4%)	46,502 (6.6%)	< 0.001
Intrauterine hypoxia	47 (1.5%)	40,760 (5.8%)	< 0.001
Other perinatal morbidity^b^	8 (0.3%)	1113 (0.2%)	0.20
*Composite Morbidity*	115 (3.6%)	94,094 (13.4%)	< 0.001
	Planned high risk at home	Planned high risk in hospital	*p* Value
	*n* = 755	*n* = 131,726	
Stillbirth	3 (4 per 1000)	370 (2.8 per 1000)	0.55
Neonatal Death	4 (5.3 per 1000)	97 (0.74 per 1000)	< 0.001
Perinatal Mortality	7 (9.3 per 1000)	467 (3.5 per 1000)	0.009
Admission to SCN	33 (4.4%)	17,995 (13.7%)	< 0.001
Admission to NICU	12 (1.6%)	555 (0.4%)	< 0.001
Apgar< 7 @ 5 min	18 (2.4%)	2390 (1.8%)	0.24
HIE	1 (0.13%)	42 (0.03%)	0.13
Birth Trauma^a^	23 (3.1%)	10,056 (7.6%)	< 0.001
Intrauterine hypoxia	24 (3.2%)	8714 (6.6%)	< 0.001
Other perinatal morbidity^b^	3 (0.4%)	287 (0.2%)	0.29
*Composite Morbidity*	59 (7.8%)	22,223 (16.9%)	< 0.001

^a^Birth trauma includes brachial plexus injury, fractured clavicle or humerus

^b^Other perinatal morbidity includes meconium aspiration syndrome, congenital pneumonia or respiratory distress syndrome

For high risk women there was no statistically significant difference in the rate of stillbirth between women who planned to birth at home compared to those who planned to birth in hospital but the rate of neonatal death was 7.2-fold higher (5.3 per 1000 v’s 0.74 per 1000, *p* < 0.001) and the rate of NICU admission was 4-fold higher (1.6% v’s 0.4%, *p* < 0.001). In contrast, the rates of admission to SCN (4.4% v’s 13.7%, *p* < 0.001), birth trauma(3.1% v’s 7.6%, *p* < 0.001), intrauterine hypoxia(3.2% v’s 6.6%, *p* < 0.001) and composite perinatal morbidity(7.8% v’s 16.9%, *p* < 0.001) were significantly lower in the planned homebirth group. There were no statistical differences in low (< 7) 5 min Apgar score, HIE or other perinatal morbidity between the groups.

Risk factors identified in the perinatal deaths among high risk planned hospital birth were “maternal medical complications” (35% of the perinatal deaths), previous caesarean (28% of the perinatal deaths); non-cephalic presentations (22% of the perinatal deaths), twin pregnancies (11% of the perinatal deaths) and a BMI of over 35 (16% of the perinatal deaths). Multiple risk factors existed for women in hospital with past caesarean, maternal obesity and maternal medical conditions frequently co-existing. In regards to women at home past caesarean and being post term were the most common risk factors.

### Maternal and obstetric outcomes (Table 4)

There was one maternal death among women who planned to give birth at home and 23 among those who planned to give birth in hospital.

**Table 4:** Rates of adverse maternal outcomes and obstetric interventions among planned home and hospital births

	Planned low risk at home	Planned low risk in hospital	*p* Value
	*n* = 3202	*n* = 701,058	
Mother outcomes			
*Composite maternal morbidity*	344 (10.7%)	121,248 (17.3%)	< 0.001
Mother admitted to HDU/ICU	6 (0.2%)	4217 (0.6%)	0.002
3rd/4th degree tear	31 (1.0%)	14,000 (2.0%)	< 0.001
Postpartum haemorrhage	291 (9.1%)	90,603 (12.9%)	< 0.001
Blood Transfusion	10 (0.3%)	5174 (0.7%)	0.005
Manual removal of placenta	29 (0.9%)	17,354 (2.5%)	< 0.001
Rupture of uterus	0 (0 per 1000)	94 (0.13 per 1000)	0.5
Intrapartum haemorrhage	3 (0.1%)	2565 (0.4%)	0.01
Obstetric Interventions			
Unplanned Caesarean	81 (2.5%)	87,716 (12.5%)	< 0.001
Instrumental delivery	79 (2.5%)	122,517 (17.5%)	< 0.001
Epidural	101 (3.2%)	192,821 (27.5%)	< 0.001
General Anaesthetic	5 (0.2%)	6754 (1.0%)	< 0.001
Episiotomy	92 (2.9%)	148,868 (21.2%)	< 0.001
Spontaneous Vaginal	3038 (94.9%)	490,748 (70.0%)	< 0.001
	Planned high risk at home	Planned high risk in hospital	*p* Value
	*n* = 743	*n* = 128,228	
Mother outcomes			
*Composite maternal morbidity*	124 (16.7%)	31,543 (24.6%)	< 0.001
Mother admitted to HDU/ICU	4 (0.5%)	1459 (1.1%)	0.06
3rd/4th degree tear	12 (1.6%)	2443 (1.9%)	0.58
Postpartum haemorrhage	108 (14.5%)	25,079 (19.6%)	< 0.001
Blood Transfusion	14 (1.9%)	1634 (1.3%)	0.25
Manual removal of placenta	15 (2.0%)	4058 (3.2%)	0.047
Rupture of uterus	1 (1.3 per 1000)	138 (1.1 per 1000)	0.37
Rupture of uterus (women with past caesarean only)	1 (3.2 per 1000)	138 (3.6 per 1000)	0.89
Intrapartum haemorrhage	3 (0.4%)	673 (0.52%)	0.65
Obstetric Interventions			
Unplanned Caesarean	66 (8.9%)	41,530 (32.4%)	< 0.001
Instrumental delivery	34 (46%)	18,407 (14.4%)	< 0.001
Epidural	40 (5.4%)	35,266 (27.5%)	< 0.001
General Anaesthetic	4 (0.5%)	3450 (2.7%)	< 0.001
Episiotomy	30 (4.0%)	20,329 (15.9%)	< 0.001
Spontaneous Vaginal	640 (86.1%)	68,219 (53.2%)	< 0.001

Low risk women who planned a home birth were significantly more likely to have a spontaneous vaginal birth(94.9% v’s 70%, *p* < 0.001). For low risk women, planned home birth was associated with a significantly lower rate of maternal HDU/ICU admission (0.2% v’s 0.6%; *p* = 0.002), severe perineal trauma (1.0% v’s 2.0%; *p* < 0.001), postpartum haemorrhage(9.1% v’s 12.9%; *p* < 0.001), blood transfusion(0.3% v’s 0.7%; *p* = 0.005), manual removal of placenta(0.9% v’s 2.5%; *p* < 0.001) and intrapartum haemorrhage(0.1% v’s 0.4%; *p* = 0.01). The rates of unplanned caesarean (2.5% v’s 12.5%; < 0.001), assisted vaginal birth(2.5% v’s 17.5%; *p* < 0.001), epidural analgesia(3.2% v’s 27.5%; *p* < 0.001), general anaesthetic(0.2% v’s 0.96%; *p* < 0.001), and episiotomy(2.9% v’s 21.2%; *p* < 0.001) were significantly lower in those women planning to give birth at home.

High risk women who planned a home birth were also significantly more likely to have a spontaneous vaginal birth than those who planned a hospital birth (86.1% v’s 53.2%; *p* < 0.001). Among high risk women, planning to birth at home was associated with a significantly lower rate of postpartum haemorrhage(14.5% v’s 19.6%; *p* < 0.001) and composite maternal morbidity (a combination of all maternal morbidities combined) (16.7% v’s 24.6%; *p* < 0.001) than planned hospital birth. Obstetric interventions were also significantly less common among the high risk women who had planned a home birth compared to those who planned a hospital birth: unplanned caesarean(8.9% v’s 32.4%; < 0.001), assisted vaginal birth(4.6% v’s 14.4%; *p* < 0.001), epidural(5.4% v’s 27.5%; *p* < 0.001), general anaesthetic(0.5% v’s 2.7%; *p* < 0.001), and episiotomy(4.0% v’s 15.9%; *p* < 0.001).

## Discussion

Overall we found that home birth, regardless of risk status, was associated with significantly lower rates of obstetric interventions and combined overall maternal and perinatal morbidities. For low risk women planned homebirth was also associated with similar risks of perinatal mortality, however for women with recognized risk factors, planned homebirth was associated with significantly higher rates of perinatal mortality.

Until now, the outcomes from home births in Victoria, particularly among “high risk” women was unknown. We found that for women with risk factors home birth was associated with a significantly increased risk of neonatal death and some perinatal morbidities. Our findings broadly accord with those of the Birthplace in England study that showed that planned homebirth among “high risk” women was associated with a 2-fold increased risk of a composite perinatal outcome (intrapartum stillbirth, early neonatal death, hypoxic ischaemic encephalopathy, meconium aspiration syndrome, brachial plexus injury, fractured clavicle or humerus and admission to NICU for greater that 48 h), albeit that increase was not statistically significant [12]. The Birthplace study also reported a moderate (1.20 95% CI 0.41–3.44) but non-significant increase in risk of adverse perinatal outcomes among a small number of women planning a VBAC at home compared to in hospital [13]. Not all adverse perinatal outcomes were increased in the planned homebirth group however. High risk women planning to birth at home experienced significantly reduced rates of birth trauma and intrauterine hypoxia. We also observed that high risk women who planned to birth at home experienced lower rates of maternal morbidities.

Qualitative studies have been undertaken to understand why women with certain risk factors, particularly those who have had a previous caesarean choose to birth at home. These studies identify a recurrent theme that women want to avoid another caesarean [16]. Our results appear to vindicate their fears. In Victoria, high risk women who planned to birth in hospital had over 3.5 times the rate of unplanned caesarean than those who planned to birth at home. We were not able to address whether this difference was clinically justified or not. However, recently, Dutch investigators aimed to determine if the differences in intervention rates between home and hospital births reflected over or under treatment in these groups [17]. They found both. Our findings here and the observation that there is 10-fold variation in the rate of attempted VBAC rates in maternity units in Victoria [18] would suggest the same is occurring here. There is an urgent need for health services to reassess their policies and support for women planning a VBAC to address these discrepancies in care. Previous research has also identified that women with risk factors also choose to birth at home, do so following a traumatic or bad experience in hospital [19]. Addressing discrepancies in care, and providing support and understanding the experiences of women following adverse pregnancy and labour experiences after giving birth in hospital is needed. Interestingly, despite much debate around absolute risk [20], uterine rupture is commonly cited as a reason that women who have had a previous caesarean should not plan a vaginal birth [21]. The only uterine rupture that occurred in the high risk women who planned a home birth was in a woman who had had more than one previous caesarean section.

Reporting the outcomes for low risk women planning a home birth was necessary so that if we found higher rates of adverse outcomes for high risk women, as we did, we could consider whether this was a feature of home birth per se in Victoria. This was not the case. Perinatal outcomes from planned homebirth in low risk women were similar to those of planned hospital birth. The low risk women planning a home birth also had fewer obstetric interventions and lower rates of maternal morbidities. Our findings were consistent with other studies of low risk women giving birth at home [1].

Seventeen percent of high risk and 10% of low risk women required transfer to hospital. Women and/or babies requiring a transfer to hospital in labour are considered at most risk of adverse outcomes [22]. We did not find that. Instead, we found that the majority of adverse perinatal outcomes were among the women who actually gave birth at home. Understanding why this is the case is needed. A recent survey of intrapartum transfers with 13 Australian midwives identified barriers to timely transfer of women to hospital [23]. Midwives reported health services either refusing to accept transfers or health services/hospital staff being hostile to the midwives when they arrived [23]. This was in contrast to experiences some of these midwives had when they worked as a homebirth midwife in the UK [23]. The rate of homebirth in Australia is much lower than the rest of the world. In Australia only 0.3% of women plan to give birth at home. This is in contrast to 3.4% of women in New Zealand, 2% in Canada and the UK, and 20% in the Netherlands. The organisation of homebirth in Australia also differs. In Victoria the majority – almost three quarters –of women having a homebirth do so under the care of a private midwife. This differs from the UK, Netherlands and New Zealand where homebirth is integrated and offered within the public health care system.

Our study had a number of limitations. Prior to 2009 maternal BMI were not reported in the dataset. It is possible that low risk women were actually of high risk prior to 2009. The adverse findings for the low risk women may therefore be overestimated and for the high risk women underestimated. Due to small numbers of adverse outcomes we were unable to stratify our results by parity. There is also the potential for misclassification of planned place of birth and risk status. It is possible that some women with risk factors indicated they planned to give birth in hospital when in fact they planned to birth at home or changed their intent and did not notify the hospital. We were also unable to link to past obstetric complications that would identify a woman as high risk e.g. previous postpartum haemorrhage. It is possible that the denominator for planned homebirth for high risk women is larger than we have reported*.* Furthermore it is possible that differences in fetal monitoring that occur at home (intermittent auscultation) compared to hospital (continuous CTG and possibly fetal scalp lactates) and health service preference to directly admit babies to the NICU rather than SCN may have led to an underestimation of the rates of intrauterine hypoxia and SCN admission in the planned homebirth group. This likely explains the discordance between higher rates of NICU admission, but lower rates of perinatal morbidity. Caution should be made interpreting these findings in isolation.

## Conclusions

We report the rates of selected perinatal, maternal and obstetric outcomes in Victorian women with and without risk factors, by their planned place of birth. Our findings support current guidelines [14, 24–26] and should be useful to women who are making informed choices about where to have their baby and to health services and policy advisors who are planning the provision of maternity care.
